# Novel Approach for Glycemic Management Incorporating Vibration Stimulation of Skeletal Muscle in Obesity

**DOI:** 10.3390/ijerph20064708

**Published:** 2023-03-07

**Authors:** Mijin Kim, Hanlin Zhang, Taeho Kim, Yutaro Mori, Tomohiro Okura, Kiyoji Tanaka, Tomonori Isobe, Takeji Sakae, Sechang Oh

**Affiliations:** 1 Institute of Food Research, National Agricultural and Food Research Organization, Tsukuba 305-8642, Japan; 2 R&D Center for Tailor-Made QOL, University of Tsukuba, Tsukuba 305-8550, Japan; 3 Graduate School of Comprehensive Human Sciences, University of Tsukuba, Tsukuba 305-8572, Japan; 4 The Center for Sports Medicine and Health Sciences, Tsukuba University Hospital, Tsukuba 305-8576, Japan; 5 Faculty of Medicine, University of Tsukuba, Tsukuba 305-8575, Japan; 6 Faculty of Health and Sport Sciences, University of Tsukuba, Tsukuba 305-8572, Japan; 7 Faculty of Rehabilitation, R Professional University of Rehabilitation, Tsuchiura 300-0032, Japan

**Keywords:** vibration therapy, obesity, impaired glucose tolerance, myokine, muscle stiffness

## Abstract

Because obesity is associated with impaired glucose tolerance and type 2 diabetes (T2D), it is important to manage the blood glucose level at an early stage. Nevertheless, people with obesity have significantly lower resistance to muscle fatigue after exercise and exercise adherence. Therefore, we developed a novel “Relaxing-Vibration Training (_R_VT)” consisting of 25 postures using vibration stimulation of skeletal muscle and determined the feasibility of _R_VT for glycemic management. Thirty-one participants with obesity were enrolled in a controlled trial (CT) and experimental trial (ET) based on a 75 g oral glucose tolerance test (OGTT). During the CT, participants were required to rest in a quiet room. During the ET, the _R_VT program (50 Hz, 4 mm), consisting of 25 postures of relaxation and stretching on the vibratory platform, was performed for 40 min. Subsequently, the participants rested as in the CT. Subjective fatigue and muscle stiffness measurements and blood collection were conducted before and after _R_VT. In both the CT and ET, interstitial fluid (ISF) glucose concentrations were measured every 15 min for 2 h. The incremental area under the curve value of real-time ISF glucose during an OGTT was significantly lower in the ET than in the CT (ET: 7476.5 ± 2974.9, CT: 8078.5 ± 3077.7, effect size r = 0.4). Additionally, the levels of metabolic glucose regulators associated with myokines, muscle stiffness, and subjective fatigue significantly improved after _R_VT. This novel _R_VT suggests that it is effective in glycemic management with great potential to improve impaired glucose tolerance and T2D with obesity in the future.

## 1. Introduction

The increasing prevalence of obesity directly and indirectly contributes to increased morbidity and mortality, including type 2 diabetes (T2D) [1]. Considering that impaired glucose tolerance (IGT) and T2D are paradigms of obesity-related disorders, it is necessary to manage the blood glucose metabolism in people with obesity at an early stage. Exercise and diet restriction have been recommended as crucial preventive treatment measures. Exercise is recognized to be more effective in the prevention and improvement of insulin resistance and T2D in the long term because it increases mitochondrial biogenesis and improves glucose tolerance and insulin action [2]. However, people with obesity have significantly lower muscle fatigue resistance after exercise than those with non-obesity [3]. Additionally, patients with T2D have greater muscle fatigability in both lower and upper body muscles [4]. Even though exercise is effective in regulating glucose metabolism, most people with obesity maintain a sedentary lifestyle, and exercise adherence is low because of physical limitations, musculoskeletal discomfort, and physical and psychological fatigue [5]. Therefore, there is a need for an alternative to conventional exercise that is relaxing, safe, easy to continue, and effective.

Recently, vibration training (VT), which contracts and relaxes skeletal muscles by vibration stimulation without a mass load or dynamic exercise, has received considerable attention. This mechanical stimulation uses proprioceptive spinal reflexes to induce an amyotrophic stretch reflex mediated by the muscle spindle and a type-Ia sensory fiber, thereby facilitating activity of homonymous α-motor neurons [6]. It has been proved that this technique has a similar benefit to resistance exercise based on voluntary muscle contraction [7]. VT is effective in plasma glucose regulation because it increases glucose utilization by causing voluntary muscle contraction through vibration stimulation [8]. In addition, it has been reported to be effective in improving glycemic indicators and lipid-related cardiovascular risk factors [9]. However, most VT was designed as an alternative to resistance exercise movements such as squats and lunges, which are accompanied by muscle pain, and fatigue, as we have previously experienced [10,11]. Therefore, the former approach may also be unsuitable for those who do not favor exercise and those with limitations in mobility and posture among people with obesity.

On the basis of these considerations, in this pilot study, we aimed to advance a previously general VT that requires high-intensity resistance posture and developed a novel “Relaxing Vibration Training (_R_VT)” program that can be performed more comfortably and safely. As a first stage, we conducted an acute trial to verify the feasibility of a novel _R_VT for glycemic management. Using interstitial fluid (ISF) glucose concentrations during an oral glucose tolerance test (OGTT), blood markers, and muscle stiffness and fatigue were determined in middle-aged and older adults with obesity. We hypothesized that our practice of _R_VT has a more positive effect on subjective and biochemical indicators and has potential viability as a glycemic management program. The primary outcome was a change in the ISF glucose concentration, and the secondary outcomes were a change in blood markers, muscle stiffness, and fatigue.

## 2. Materials and Methods

### 2.1. Ethical Approval and Study Design

**Figure 1:**
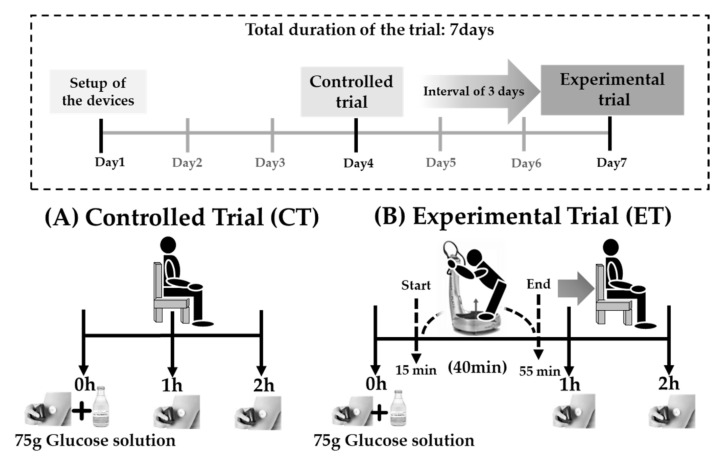
Schematic illustrating the experimental protocol and time course of the study in (**A**) the CT and (**B**) the ET.

This study of a single-arm, acute intervention design was conducted for 7 days (March 2021) at the University of Tsukuba. The study objectives, design, criteria of inclusion and exclusion, assessments, practice of _R_VT, OGTT, insurance compensation for injury, withdrawal of consent, and privacy protection were explained face-to-face to eligible participants. Written informed consent was obtained from each participant, the study was conducted in accordance with the Declaration of Helsinki and was approved by the ethical committee of the University of Tsukuba (reference no. Tai 020-95). The study protocol was registered at the University Hospital Medical Information Network center (UMIN no. 000042787). We applied the devices, the Free-Style Libre Flash continuous glucose monitoring (FSL-CGM) system (Abbott Diabetes Care, Witney, UK) and the Polar A370 fitness tracker (Polar Electro Oy, Kempele, Finland), to study the participants’ bodies on the first day. Thereafter, the controlled trial (CT) was conducted on the fourth day and the experimental trial (ET) on the seventh day. Participants were required to fast for more than 10 h before each trial and were forbidden to consume alcohol or perform excessive exercise on the day before the trials. In both trials, participants ingested 225 mL soda-flavored solution (TRELAN^®^ G75; Ajinomoto Pharmaceuticals Co., Ltd., Tokyo, Japan) containing 75 g glucose and performed an OGTT lasting for 2 h, the ISF glucose concentration being recorded every 15 min. The details of the CTs and ETs are as follows (Figure 1): 

#### 2.1.1. CT

Participants were asked to fill out a questionnaire consisting of questions about age, sex, smoking, alcohol intake, and medical history, and to measure blood pressure and heart rate (OMRON HEM-7111, Kyoto, Japan). Additionally, during the 2 h OGTT, participants were required to rest while measuring ISF glucose concentrations every 15 min on a chair in a quiet room. They were allowed to perform tasks such as reading a book, watching a movie, or operating a computer.

#### 2.1.2. ET

Participants performed _R_VT for 40 min, starting 15 min after the intake of a 75 g glucose solution. Subjective fatigue surveys, muscle stiffness measurements, and blood collection were conducted before and after _R_VT. Subsequently, the participants moved to a quiet room and rested as in the CT. In this study, we used a vibration machine (Pro5 AIRdaptive; Power Plate, Badhoevendorp, The Netherlands) that can deliver three-dimensional harmonic vibration to the body. An expert with a power plate instruction certificate developed a novel _R_VT consisting of 25 postures of relaxation and stretching. The _R_VT was performed for 1 min per posture with a frequency of 50 Hz and an amplitude of 4 mm for 40 min on the vibratory platform, including preparation time for the next posture and rest (Figure S1).

### 2.2. Participants

**Figure 2:**
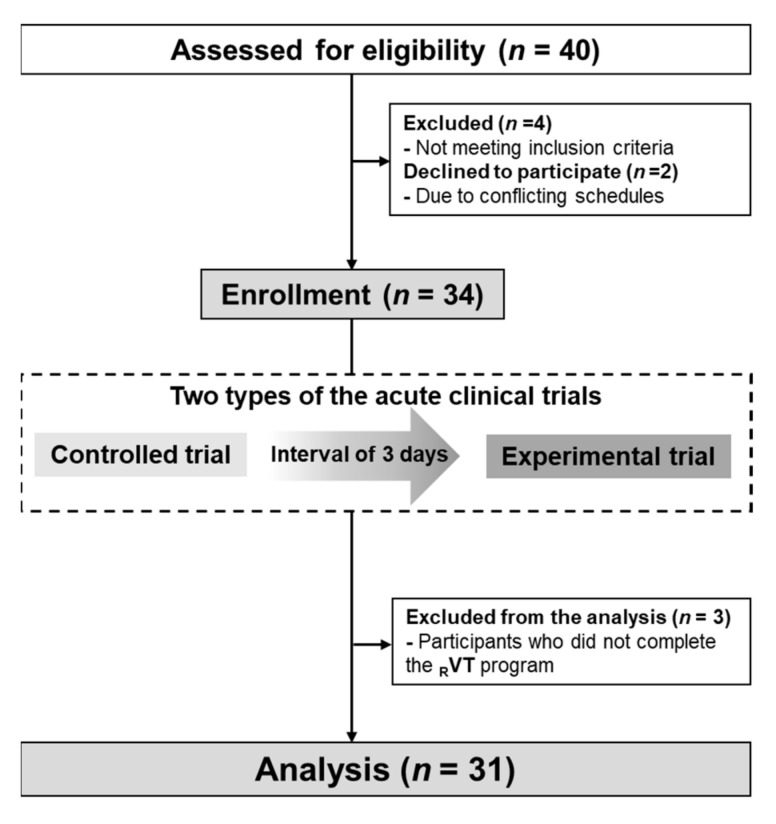
Flow diagram for the study.

On estimating the sample size using the statistical software G* Power 3.1, 34 subjects were required as the total sample size (a priori effect size = 0.5, α = 0.05, power [1 − β] = 0.8). Forty participants with obesity residing in Tsukuba City, Japan were recruited through snowball sampling and a regional information magazine (Joyo Living Co., Ltd., Tsukuba, Japan). A screening survey via telephone or face-to-face interviews was conducted using a self-reported questionnaire. The inclusion criteria were: (1) age ranging over 40–74 years, (2) body mass index (BMI) ≥ 25 kg/m^2^, (3) having one or more risk factors for metabolic syndrome, (4) active participation in the study. The participants were excluded if they (1) took neuropsychiatric drugs, (2) were prohibited from exercising by doctors due to serious diseases, including brain dysfunction, renal disease, liver dysfunction, heart disease, and peripheral angiopathy, (3) had an excessive alcohol intake (>60 g/day) [12], (4) had participated in other clinical studies within the past three months, (5) were pregnant or possibility pregnant, (6) were judged inappropriate by the lead principal investigator, for example, conduct that interferes with the progress of the research by not cooperating with the research, making a fuss, or fighting with other participants. In total, 40 people with obesity applied for this study. However, four applicants were excluded according to the criteria and two applicants declined to participate because of conflicting schedules. Additionally, the participants experienced _R_VT for approximately 5 min to check whether there were any problems on the body and whether it was feasible. We finally analyzed data from 31, excluding 3 participants who did not complete _R_VT for 40 min due to personal reasons (a posteriori effect size = 0.5, α = 0.05, power [1 − β] = 0.8) (Figure 2).

### 2.3. ISF Glucose Concentrations

The glucose concentration was determined using the FSL-CGM system, which has the advantage of being able to measure ISF glucose concentrations in real-time without blood collection through a sensor attached to the subcutaneous tissue. The FSL-CGM system consists of a glucose reader and sensor and the activation time for the sensor is 14 days, with the sensor’s high accuracy and convenience having been shown in previous studies [13,14]. Before the study, a health professional attached a sensor of the FSL-CGM to the rear upper arm of the participants under an aseptic technique and monitored it for 48 h to confirm the adaptability and stability of the ISF glucose concentration measurement. Participants were asked to set the alarm for the 2 h OGTT in both trials and measure the ISF glucose concentrations by touching the reader to the sensor every 15 min and recording the value in the datasheet.

### 2.4. Characteristics of Participants 

BMI (kg/m^2^) was calculated as body weight in kilograms divided by height in meters squared. Fat and muscle mass were determined using a bioelectrical impedance analyzer (MC-980A, TANITA, Tokyo, Japan). We measured the waist circumference (WC), blood pressure, fasting blood glucose, high-density lipoprotein cholesterol (HDL-C), and triglyceride (TG) to confirm whether the participants displayed the corresponding risk factors for metabolic syndrome. Risk factors for metabolic syndrome were evaluated according to the Japanese standards as follows: abdominal obesity (WC: male ≥ 85 cm, female ≥ 90 cm), high blood pressure (systolic blood pressure ≥ 130 mmHg, diastolic blood pressure ≥ 85 mmHg, or a history of hypertension), high fasting glucose (≥110 mg/dL or a history of diabetes), low HDL-C (<40 mg/dL), and hypertriglyceridemia (≥150 mg/dL or a history of hyperlipidemia) [15]. To measure the amount of daily physical activity (PA) and sleep time, participants were required to wear a Polar A370 fitness tracker based on a wrist-worn three-axis accelerometer model for the entire study period of 7 days [16]. The number of walking steps, total sleep time, and PA time by intensity (light, moderate, vigorous) were divided into baseline and during the experiment, and the mean values of each were calculated.

### 2.5. Blood Markers

Blood samples were collected from an antecubital vein and separated fractions were stored at −80 °C until further analysis. The free fatty acids (FFAs) and HDL-C levels were determined by enzyme method, lactate dehydrogenase (LDH), aspartate transaminase (AST), and creatine kinase (CK) levels by the Japan Society of Clinical Chemistry transferable method, fasting serum glucose level by the hexokinase-G-6-phosphate dehydrogenase method, high-sensitivity C-reactive protein (hs-CRP) level by fixed time assay method, and cortisol level by radioimmunoassay. We evaluated serum levels of fibroblast growth factor 21 (FGF21; R&D Systems; Minneapolis, MN, USA), interleukin 6 (IL6; R&D Systems), and myostatin (Cusabio biotech, Wuhan, China) using commercial enzyme-linked immunosorbent assay kits.

### 2.6. Muscle Stiffness and Fatigue

Muscle stiffness of the trapezius, deltoid, biceps brachii, rectus femoris, biceps femoris, tibialis anterior, and medial gastrocnemius on the dominant side was determined before and after _R_VT in the ET using the Myoton^®^ PRO (Myoton AS, Tallin, Estonia), an accurate and reliable device for non-invasive digital palpation of superficial skeletal muscles. The location of the muscle to be measured was marked, and the probe of the Myoton^®^ PRO device was vertically mounted on the surface of the measuring mark point as suggested by the manufacturer [17]. Three consecutive measurements were performed on each muscle area, and the mean value for each was used for statistical analysis. Additionally, subjective fatigue was assessed before and after _R_VT using the questionnaires “subjective symptoms of fatigue (Jikaku-sho shirabe)” and “body parts of fatigue (Hirou-bui shirabe)”, developed by the Japan Occupational Health and Occupational Fatigue Research Committee. The subjective symptoms of the fatigue questionnaire comprised 25 items divided into five categories (I: drowsiness, II: instability, III: uneasiness, IV: local pain or dullness, V: eyestrain). The body parts of the fatigue questionnaire investigated subjective fatigue levels for each body part, including the neck, shoulder, middle back, upper arm, forearm, lower back, hand, hip and thigh, lower leg and knee, and foot [18]. 

### 2.7. Statistical Analyses

Linear mixed model analysis was applied to evaluate the differences in the intervention effect of the two trials on the change of ISF glucose concentrations. On the basis of significant interactions (trials × times), this study performed post hoc analysis with Bonferroni correction. Wilcoxon signed-rank test was used only in the ET to compare changes in the incremental area under the curve (IAUC) of the ISF glucose concentration response, blood markers, muscle stiffness, and fatigue before and after _R_VT practice. The IAUC value is the sum of the areas of triangles and rectangles geometrically calculated using the elapsed time from baseline to 120 min and the response value of ISF glucose concentration. On the basis of the fasting blood sugar (0 min), the sum of increased values at 15 min (A), 30 min (B), 45 min (C), and 60 min (D; divided by 2) were multiplied by 15 and the sum of the increased values at 90 min (E) and 120 min (F; divided by 2) were multiplied by 30 {IAUC = (A + B + C + (D/2)) × 15((D/2) + E + (F/2)) × 30} [19]. To verify the effect sizes (0.1: small, 0.3: medium, 0.7: large) of all variables from the baseline to after the _R_VT, the effect size r was calculated as the Z statistic divided by the square root of the sample size (N) (Z/√N). All statistical analyses were performed using SPSS version 26.0 (IBM Corp., Armonk, NY, USA), with significance levels set to *p* < 0.05. The rate of change (Δ (%)) was calculated by subtracting the baseline value from the value after _R_VT practice and was expressed as a percentage of the baseline value. 

## 3. Results

### 3.1. Characteristics of Participants

As shown in Table 1, the age of the 31 participants (48.4% female, 51.6% male) was 55.6 ± 8.4 yr. All participants had abdominal obesity, and it could be inferred that the participants were of the type with much muscle mass (49.5 ± 11.2 kg) but also considerable fat mass (male: 25.3 ± 7.6 kg, female: 30.3 ± 9.5 kg). Additionally, there were no significant changes in the sleep time, walking, or PA during the experiment period (Table S1).

### 3.2. Comparison of ISF Glucose Concentrations by the OGTT in Two Trials

**Figure 3:**
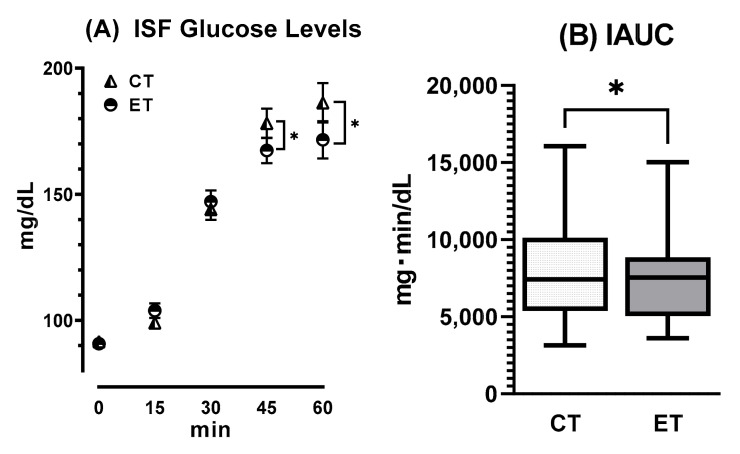
Comparison of (**A**) ISF glucose concentrations and (**B**) the IAUC in the CT and the ET. Data are expressed using (**A**) the linear mixed model (mean ± SE) and (**B**) the Wilcoxon signed-rank test (median, min, max), * *p* < 0.05. Abbreviations: CT: controlled trial, ET: experimental intervention trial, ISF: interstitial fluid, IAUC: incremental area under the curve, SE: standard error.

Figure 3A shows a comparison of the changes in ISF glucose concentrations with time of the two trials, focusing on within 1 h of the OGTT and before and after _R_VT practice. A significant interaction (*p* = 0.014) was observed between ISF glucose concentrations and the two trials, and according to the post hoc analysis, the ISF glucose concentrations in the ET was significantly reduced at 45 min (*p* = 0.019) and 60 min (*p* = 0.001). Figure 3B shows the comparison of the IAUC of ISF glucose concentrations of the two trials during 2 h of the OGTT, with the ET being significantly lower than the CT (*p* = 0.047).

### 3.3. Changes in Blood Markers after _R_VT

**Figure 4:**
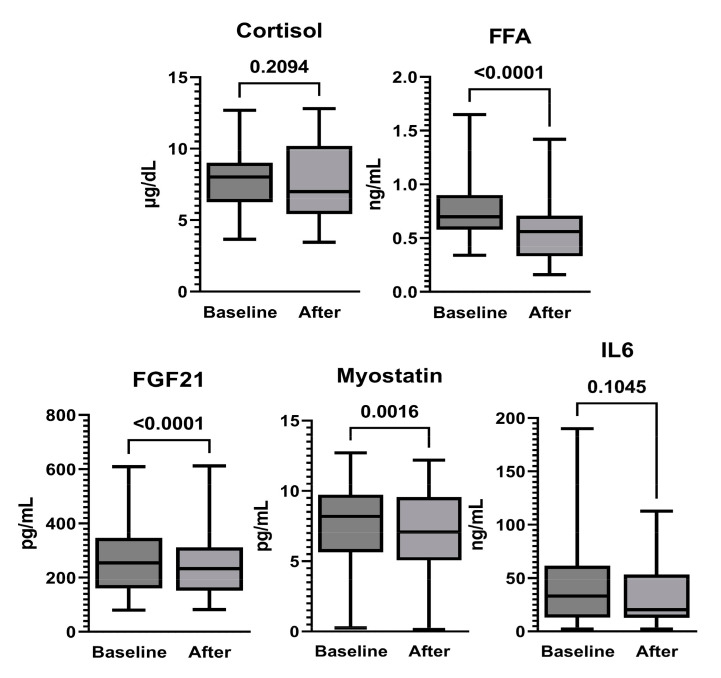
Changes in the levels of markers of metabolic regulation of glucose and lipid after _R_VT: cortisol, FGF21, myostatin, IL-6, and FFAs. Data are expressed using the Wilcoxon signed-rank test (median, min, max). Abbreviations: FGF21: fibroblast growth factor 21, IL6: interleukin 6, FFA: free fatty acid.

FGF21 and myostatin as markers of metabolic glucose regulation were significantly decreased (Figure 4). FFAs as relative marker of lipid utility was significantly decreased (Figure 4). Additionally, there were no changes in the muscle-damage markers CK, AST, LDH, or hs-CRP after _R_VT (Table 2).

### 3.4. Changes in Muscle Stiffness and Fatigue after _R_VT

The stiffness of the medial gastrocnemius was significantly decreased (*p* < 0.05, *r* = −0.43). Regarding the fatigue of body parts, the upper-body parts (*p* < 0.01, *r* = −0.65), lower-body parts (*p* < 0.01, *r* = −0.63), and total score of the whole body (*p* < 0.01, *r* = −0.71) decreased significantly. Additionally, drowsiness (*p* < 0.01, *r* = −0.51), instability (*p* < 0.05, *r* = −0.42), local pain or dullness (*p* < 0.01, *r* = −0.71), eyestrain (*p* < 0.01, *r* = −0.64), and total score of fatigue symptoms (*p* < 0.01, *r* = −0.70) decreased significantly after _R_VT (Table 2).

## 4. Discussion

Vibration stimulation of skeletal muscles and tendons activates muscle spindles, which is termed “tonic vibration reaction”. It activates more motor neurons, which in turn activates motor units and the movement of actin and myosin, increasing muscle contraction [20]. Such muscle contraction increases the content of glucose transporter 4 in muscle cells and its translocation to the sarcolemmal membrane, which considerably improves glucose transport capacity [21,22]. As such, muscle activation aids in glucose and insulin control, thus it has been actively suggested for T2D patients. Therefore, for those people with obesity who have a sedentary lifestyle or the elderly with weak joints and strength, muscle contraction by the tonic vibration reaction is considered as a more effective treatment. A meta-analysis study reported that VT interventions could reduce fasting blood glucose concentrations by 25.7 mL/dL in older adults with T2D [23]. It has been reported that 12 weeks of VT and strength training decreased the IAUC values of the OGTT [24], and that glycosylated hemoglobin values improved after VT for 6 [25] and 12 weeks [24]. By contrast, a previous study that performed acute VT reported reduced blood glucose concentrations in both diabetic patients and healthy elderly women [26]. In our pilot study, designed as an acute intervention, ISF glucose concentrations were significantly lower with respect to the IAUC values of the 2 h OGTT as well as during the practice of _R_VT (45 min) and immediately after the end of _R_VT (60 min). Overall, our study supports the positive result of previous studies that the glucose concentration can be controlled regardless of whether the period of skeletal muscle vibratory stimulation is acute or chronic. 

When muscle tissue is stimulated, it secretes myokines, which affect inflammation, glucose processing, and adipose tissue. FGF21 and myostatin, myokine factors, are known to be associated with obesity and insulin resistance [27]. It was reported that FGF21 is involved in glucose metabolism regulation and promotes blood glucose absorption by adipocytes [28]. However, “paradoxical” plasma FGF21 elevation in obesity and diabetes suggests a potential FGF21-resistant state [29]. Additionally, myostatin increases with obesity and with a lack of exercise, which is involved in the acquisition of insulin resistance [30]. Therefore, a balanced FGF21 level and a reduced myostatin level are also attracting attention as potential therapeutic targets for insulin resistance in T2D. In older men, the intervention effect of resistance exercise decreased FGF21 and myostatin and increased muscle strength in both T2D and non-T2D subjects [31]. After performing _R_VT in the present study, there were great decreases in the ISF glucose concentration, FGF21, and myostatin. Contrary to the results of this study, it was reported that acute VT in both overweight and normal weight subjects displayed a time-dependent response in IL6, glucose, and insulin, but no change in myostatin [32]. By contrast, myostatin mRNA expression started to decrease 1 h after resistance exercise and was most suppressed after 8 h, while IL6 expression was highest after 4 h [33]. In the previous studies above, it was shown that glucose metabolism and myokines react in response to muscle contraction, whether voluntary or involuntary; however, opinions are still divided on the reactions as time course and the physical interaction. Also, FFAs as a relative marker of lipid utility were significantly decreased. The _R_VT may stimulate the hydrolysis of TG, which results in the release of FFAs to circulation and their oxidation in skeletal muscles. The elevated blood lipid level, observed in obesity and metabolic syndrome, is an adverse condition that leads to lipotoxicity and ectopic fat deposition in other organs, and consequently insulin resistance and impaired glucose metabolism [34]. Thus, efficient uptake and oxidation of FFAs in working muscles by intense vibration may be effective for retracting their high blood glucose levels.

Moreover, because vibration stimulation of skeletal muscle has different physiological effects depending on the parameter settings (e.g., frequency, amplitude, posture), the protocol should be carefully set by a certified professional [35]. In a previous study, VT was set at 12–16 Hz frequency, 4 mm amplitude, and resistance exercise [9], and in another study, it was set at 30 Hz frequency, 2 mm amplitude, and squat posture [36]. Both studies showed a significant decrease in fasting blood glucose in the VT group compared with the control group. Compared with the protocols of the previous studies (12–30 Hz, 2–4 mm), that of the present study (50 Hz, 4 mm) was set a higher, but was consistent with the fact that vibration stimulation of skeletal muscle can modulate glucose concentration. Hazell et al. reported that the greater the amplitude (4 mm) and frequency (35, 40, 45 Hz) of VT, the greater the measured electromyography activity of the muscle in both static and dynamic contractions [37]. It was also suggested that the frequency should not be <20 Hz to avoid the resonance frequency range [35]. Despite the high frequency and amplitude, no adverse events were reported from the participants during _R_VT, rather, the results that the muscle stiffness and fatigue improved showed satisfaction with this _R_VT. 

More importantly, we need to focus on the specific postures that were performed on the vibrating platform. High-intensity postures such as resistance exercise are difficult protocols for the elderly or diabetic patients in the high-frequency and high-amplitude settings. Because people with obesity and T2D have lower exercise adherence than healthy adults, strenuous resistance exercise accompanied by muscle fatigue is more likely to reduce exercise continuity [3,4]. The novel development of _R_VT in the present study significantly improved medial gastrocnemius stiffness, drowsiness, instability, local pain or dullness, eye strain, and whole-body fatigue. Moreover, after _R_VT practice in this study, the muscle-damage markers CK, AST, LDH, and hs-CRP did not change. In the participants with fibromyalgia, VT exercise (30 Hz, 2 mm) and conventional exercise significantly reduced pain and fatigue compared with the control group; however, stiffness and depression did not change. Additionally, it was insufficient to prove the effect of VT alone [38]. Another previous study reported that an acute VT performed for 60 sec in a half-squat posture on a vibrating platform set at 35 Hz and 5 mm was effective in reducing delayed-onset muscle soreness, the pressure pain threshold, and CK in young adults [39]. Delayed-onset muscle soreness after strenuous exercise such as resistance exercise and sprint were reduced by 22–61% after muscle massage with five stretching movements of lower extremities on a vibrating machine (30–50 Hz, 2 mm) [40]. Because vibration stimulation increases blood flow, it is effective in the excretion of fatigue substances. In addition, because the stiffness of the muscle is softer, the muscle is activated, such that muscle pain and fatigue are quickly recovered. Therefore, compared with the conventional exercise method, this novel _R_VT has advantages in the ability of glucose regulation and improvement of muscle fatigue while its practice is comfortable and safe.

This pilot study has several limitations. First, it did not include diabetic patients exclusively. Because it is at an early stage to verify the feasibility of a novel _R_VT, we were concerned about unexpected side effects in diabetic patients, including hypoglycemia and data instability. As mentioned in the introduction, we focused on people with obesity, because glucose regulation is important at an early stage from a prevention standpoint before the onset of IGT and T2D in obesity. Second, this pilot study was designed as an acute trial. The glucose concentration responds temporarily and sensitively to acute external stimuli such as diet and exercise. Therefore, we decided that it was necessary to first verify the novel _R_VT through an acute intervention trial. Because positive results were obtained in this situation, it is expected to show positive results in long-term interventions. Third, there was sampling bias and a relatively small sample size. Through the statistical software G* Power 3.1, 34 and 31 participants were estimated as a sample size by setting a power (1-β) of 0.8; however, it did not satisfy the sample size required for 0.95, which is the best power. However, a 1-β of statistical power indicates the possibility of Type II error (β) occurrence, with 0.8 meaning that even with 80% significant power, there is a 20% chance of not discriminating a significant difference. Additionally, it was reported that a power of ≥0.8 is more suitable for having a statistically significant difference [41]. Fourth, this study was designed as a single-arm, acute intervention, with the same person performing the CT first, followed by the ET at timed intervals. Therefore, in the next intervention study, it will be necessary to divide the participants into two groups and complete the CT and ET in a counterbalanced order. Based on results of this study, we would perform mass clinical studies exclusively on diabetic patients.

## 5. Conclusions

In this pilot study, the acute practice of the novel _R_VT improved the ISF glucose concentration, FGF21 and myostatin levels, and muscle stiffness and fatigue in middle-aged and older adults with obesity. In the future, this novel _R_VT is expected to have a positive impact as a glycemic management program that can improve glucose regulation and fatigue in people with obesity, and further improve IGT and T2D. However, because this study was the result of an acute clinical trial, it is necessary to conduct additional studies on whether the same results can be obtained when long-term intervention with this novel _R_VT is conducted in diabetic patients in the future.

## Figures and Tables

**Table 1:** Demographic features and clinical characteristics of the participants in baseline.

Parameter, Unit	
Total participants, *n*	31
Female, *n* (%)	15 (48.4)
Male, *n* (%)	16 (51.6)
Age, year	55.6 ± 8.4
Smoking, *n* (%)	8 (25.8)
Alcohol intake, *n* (%)	22 (71.0)
**Medical history**	
Hypertension, *n* (%)	12 (38.7)
Diabetes, *n* (%)	2 (6.5)
Hyperlipidemia, *n* (%)	4 (12.9)
Knee pain, *n* (%)	4 (12.9)
**Anthropometric and body composition**	
Height, cm	165.9 ± 9.4
Weight, kg	80.1 ± 13.8
BMI, kg/m^2^	29.0 ± 3.7
Muscle mass, kg	49.5 ± 11.2
Fat mass (male), kg	25.3 ± 7.6
Fat mass (female), kg	30.3 ± 9.5
**Risk factor of metabolic syndrome**	
Abdominal obesity, *n* (%)	31 (100)
High blood pressure, *n* (%)	22 (71.0)
High fasting glucose, *n* (%)	6 (19.4)
Low HDL-C, *n* (%)	1 (3.2)
Hypertriglyceridemia, *n* (%)	8 (25.8)

Abbreviations: HDL-C, high density lipoprotein cholesterol.

**Table 2:** Changes in muscle stiffness and subjective fatigue, muscle damage marker before and after the _R_VT program.

Parameter, Unit	Baseline	After	Δ (%)	*p*	*r*
**Skeletal muscle stiffness, N/m**					
Trapezius	401.8 ± 54.7	399.8 ± 64.3	−0.5	0.79	−0.05
Deltoid	273.5 ± 41.0	277.0 ± 42.0	−1.4	0.10	0.29
Biceps brachii	246.7 ± 39.2	242.0 ± 29.5	−1.3	0.75	−0.06
Rectus femoris	293.2 ± 27.9	294.5 ± 25.1	+0.6	0.41	0.15
Biceps femoris	285.0 ± 33.7	281.1 ± 31.0	−1.2	0.15	−0.26
Tibialis anterior	388.6 ± 82.2	379.8 ± 70.4	−1.5	0.26	−0.20
Medial gastrocnemius	271.0 ± 26.2	266.2 ± 20.9	−1.6	0.02	−0.43
**Body parts of fatigue, Point**					
Upper-body part	5.9 ± 5.5	1.8 ± 2.7	−34.9	<0.01	−0.65
Lower-body part	3.9 ± 3.6	1.2 ± 2.0	−41.8	<0.01	−0.63
Total score of whole-body	9.8 ± 8.5	3.0 ± 4.4	−42.8	<0.01	−0.71
**Subjective symptoms of fatigue, Point**					
Drowsiness	6.7 ± 2.3	5.5 ± 1.5	−10.7	<0.01	−0.51
Instability	5.6 ± 1.9	5.1 ± 0.4	−5.5	0.02	−0.42
Uneasiness	5.8 ± 2.0	5.2 ± 0.7	−5.2	0.12	−0.28
Local pain or dullness	8.8 ± 3.1	6.1 ± 1.5	−24.5	<0.01	−0.71
Eyestrain	6.9 ± 2.4	5.4 ± 0.9	−15.9	<0.01	−0.64
Total score of symptoms fatigue	33.8 ± 8.9	27.4 ± 3.9	−15.5	<0.01	−0.70
**Muscle damage marker**					
Creatine Kinase, U/L	147.3 ± 146.3	146.2 ± 143.8	−0.3	0.21	−0.2
Aspartate transaminase, U/L	23.5 ± 9.6	23.6 ± 9.6	+0.9	1.00	0
lactate dehydrogenase, U/L	181.0 ± 31.9	179.8 ± 30.9	−0.5	0.19	−0.24
Hs-CRP, mg/dL	0.0784 ± 0.082	0.0780 ± 0.081	−0.7	0.86	0

Note: Wilcoxon signed-rank test, Mean ± SD, Δ (%): Rate of change = (After − Baseline)/Baseline × 100. *r*: Effect size *r* = Z/$\sqrt{N}$, 0.1: small, 0.3: medium, 0.7: large. Abbreviations: _R_VT: Relaxing-Vibration Training, Hs-CRP: high-sensitive C-reactive protein.

## Data Availability

All data generated or analyzed during this study were included in this published article. In addition, upon reasonable request, the raw data supporting the findings of the study can be provided by the corresponding author.

## Supplementary Materials

The following supporting information can be downloaded at: https://www.mdpi.com/article/10.3390/ijerph20064708/s1, Figure S1: The target part of the body in the novel _R_VT program (50 Hz, 4 mm, 25 postures with relaxation and stretching), R: Right, L: Left; Table S1: Comparison of the sleep time, walking, physical activity baseline and during the experiment period; Table S2: Comparison of ISF Glucose Concentration in CT and ET within 2 h of OGTT.
