# Supplementary material for: Novel Approach for Glycemic Management Incorporating Vibration Stimulation of Skeletal Muscle in Obesity
Source: Int J Environ Res Public Health. 2023 Mar 7;20(6):4708. doi: 10.3390/ijerph20064708 (PMC10048751; doi:10.3390/ijerph20064708)
Supplement: Supplementary file 1 [file ijerph-20-04708-s001.zip › ijerph-2263274-supplementary.pdf]

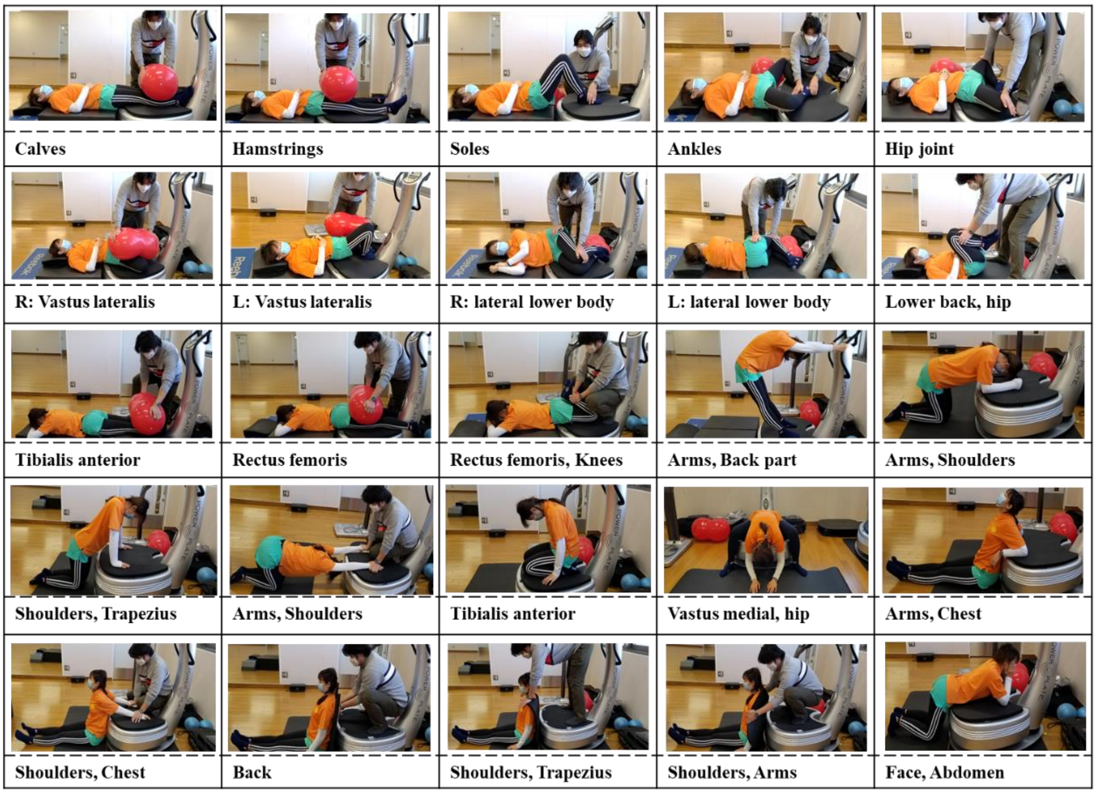


**Figure S1.** The target part of the body in the novel rVT program (50 Hz, 4 mm, 25 postures with relaxation and stretching), R: Right, L: Left

**Table S1. Comparison of the sleep time, walking, physical activity baseline and during the experiment period**

| **Parameter, unit** | **Baseline** | **During the experiment period** | ***p*** |
|---|---|---|---|
| Total sleep time, min/d | 389.0 ± 64.6 | 374.8 ± 83.0 | 0.24 |
| Walking step, step/d | 10036.2 ± 4463.4 | 10265.9 ± 3768.2 | 0.54 |
| Light PA, min/d | 335.7 ± 101.3 | 347.6 ± 126.1 | 0.51 |
| Moderate PA, min/d | 110.0 ± 71.4 | 129.3 ± 84.8 | 0.21 |
| Vigorous PA, min/d | 14.0 ± 19.1 | 15.1 ± 26.1 | 0.78 |

**Note: Wilcoxon signed-rank test, Mean ± SD, PA: physical activity, d: day**

**Table S2. Comparison of ISF Glucose Concentration in CT and ET within 2 hours of OGTT**

| Parameter, unit | Time of OGTT | CT | ET | Time effect, *P* | Trial effect, *P* | Interaction, *P* (Times × Trials) |
|---|---|---|---|---|---|---|
| ISF Glucose Concentration for 2hours, (mg/dL) | Base line 0min | 91.5 ± 7.3 | 90.7 ± 7.3 | < 0.01 | 0.02 | 0.60 |
| | 15min | 101.9 ± 7.3 | 103.9 ± 7.3 | | | |
| | 30min | 144.0 ± 7.3 | 147.1 ± 7.3 | | | |
| | 45min | 178.2 ± 7.3 | 167.4 ± 7.3 | | | |
| | 1h | 186.3 ± 7.3 | 171.6 ± 7.3 | | | |
| | 1h 15min | 184.5 ± 7.3 | 176.5 ± 7.3 | | | |
| | 1h 30min | 178.4 ± 7.3 | 170.9 ± 7.3 | | | |
| | 1h 45min | 172.5 ± 7.3 | 168.4 ± 7.3 | | | |
| | 2h | 166.6 ± 7.3 | 161.0 ± 7.3 | | | |

**Note: Liner Mixed Model, Mean ± SE, OGTT: oral glucose tolerance test, ISF: interstitial fluid, CT: controlled trial, ET: experimental trial**
